# New findings on ligand series used as SARS-CoV-2 virus inhibitors within the frameworks of molecular docking, molecular quantum similarity and chemical reactivity indices

**DOI:** 10.12688/f1000research.123550.1

**Published:** 2022-08-09

**Authors:** Alejandro Morales-Bayuelo, Jesús Sánchez-Márquez

**Affiliations:** 1 Grupo GENOMA, Escuela de Medicina, Universidad del Sinú, Cartagena, Bolivar, 3125586, Colombia; 2 Departamento de Química-Física., Facultad de Ciencias, Campus Universitario Río San Pedro, Universidad de Cádiz, Cádiz, 566842, Spain

**Keywords:** RNA dependent RNA polymerase, SARS-CoV-2 virus, COVID-19 treatments, molecular docking, molecular quantum similarity, chemical reactivity indices, density functional theory

## Abstract

**Background:** The severe acute respiratory syndrome coronavirus (SARS-CoV)-2 virus causes an infectious illness named coronavirus disease 2019 (COVID-19). SARS-CoV is a positive-sense single-stranded RNA virus from the *Betacoronavirus* genus. The SARS-CoV-2 RNA-dependent RNA polymerase (RdRp) has an important role in the viral life cycle and its active site is a very accessible region, thus a potential therapeutic approach may be to target this region to study the inhibition of viral replication. Various preexisting drugs have been proposed for the treatment of COVID-19 and the use of existing antiviral agents may reduce the time and cost of new drug discoveries, but the efficacy of these drugs is limited. Therefore, the aim of the present study was to evaluate a number of ligands used as SARS-CoV-2 virus inhibitors to determine the suitability of them for potential COVID-19 treatment.

**Methods:** In this study, we selected a series of ligands used as SARS-CoV-2 virus inhibitors such as: abacavir, acyclovir, amprenavir, ascorbic acid vitamin C, azithromycin, baloxavir, boceprevir, cholecalciferol vitamin D, cidofovir, edoxudine, emtricitabine, hydroxychloroquine and remdesivir. These ligands were analyzed using molecular docking, molecular quantum similarity, and chemical reactivity indices defined within a conceptual density functional theory framework.

**Results:** The analysis of molecular quantum similarity indices on inhibitors showed a high number of differences from a structural point of view. However, they are quite similar in their electronic density, obtaining the highest values in the electronic similarity index. Global and local chemical reactivity indices were analyzed.

**Conclusions:** These studies allowed for the identification of the main stabilizing interactions using the crystal structure of SARS-CoV-2 RdRp. The molecular quantum similarity and chemical reactivity descriptors provide novel insights into these ligands that can be used in the design of new COVID-19 treatments.

## Introduction

The severe acute respiratory syndrome coronavirus (SARS-CoV)-2 virus causes an infectious illness named coronavirus disease 2019 (COVID-19). SARS-CoV is a positive-sense single-stranded RNA virus from the *Betacoronavirus* genus. ^1^ Most RNA viruses require an RNA-dependent RNA polymerase (RdRp) for replication and transcription of the viral genome, thus making it essential for their survival, which is why it is a noteworthy topic to investigate. Its protein ranges from 240 to 450 kD and consists of a catalytic core and is a conserved protein within RNA viruses, and therefore it has been proposed as a potential option for the development of antiviral drugs. ^2^

Numerous drugs are being studied to treat COVID-19. In the USA, the first antiviral drug to treat COVID-19 in adults and children aged 12 years and older was remdesivir (Veklury). For hospitalized COVID-19 patients, remdesivir that is injected intravenously may be prescribed ( Mayo Clinic).

Molecular docking, molecular quantum similarity (MQS), and global and local reactivity indices were used in this study to evaluate remdesivir and other related compounds such as abacavir, acyclovir, amprenavir, ascorbic acid vitamin C, azithromycin, baloxavir, boceprevir, cholecalciferol vitamin D, cidofovir, edoxudine, emtricitabine and hydroxychloroquine to obtain novel insights into how the process of stabilization of these ligands at the active site in the receptor structure takes place. Previous studies have demonstrated positive responses with different levels of effectiveness for these aforementioned compounds in the treatment of COVID-19 ( Mayo Clinic).

Besalú *et al.*, introduced MQS 30 years ago to study the similarity of molecules. ^4^ The density functional theory (DFT) framework is used to connect quantum mechanics and quantum chemistry utilizing molecular docking and chemical reactivity indices. ^5^ This research is leading to advances in the discovery of new treatment alternatives for COVID-19 and determining whether approved drugs, such as remdesivir, interact with other potential ligands.

## Methods

### Methodology for docking studies

*System preparation*

The receptor structure, for the docking analysis, was extracted from the crystal structure of the SARS-CoV-2 RdRp (Protein Data Bank (PDB accession number, 6M71), which was adjusted using the Schrödinger (RRID:SCR_014879) suite 2017-1 Protein preparation Wizard (RRID:SCR_016749) module. i) The hydrogen bond network (H-bond) was optimized, and the protein structure refined, ii) at physiological pH, protonation states were determined using the PropKa utility, iii) The Impact Refinement (Impref) module was used to run a molecular minimization with heavy atoms constrained to a low root mean square deviation (RMSD) from the initial coordinates. ^6^^–^^8^

Moreover, the molecular structures of the compounds were sketched using Maestro Editor (Maestro, version 11.1, RRID:SCR_016748, Schrödinger, LLC). Then, 3D conformations were obtained with the LigPrep (RRID:SCR_016746) module, with ionization/tautomeric states predicted under physiological pH conditions with Epik (RRID:SCR_016745). Subsequently, energy minimization was used to comply with the Macro model using the OPLS2005 force field. The free alternative is AutoDock Vina (RRID:SCR_011958).

*Molecular docking*

Glide (RRID:SCR_000187) ^9^^,^^10^ with default parameters and Standard Precision (SP) model were used for docking investigations. The docking grid was created using default settings, with the co-crystallized ligand in the center. For the van der Waals radii of the nonpolar protein atoms, a scaling factor of 0.8 was applied to facilitate the binding of larger ligands. Extra precision (XP) was also utilized to expand alternate receptor conformations appropriate for binding ligands with unusual orientations *via* induced fit docking (IFD); this method allows the protein to undergo side-chain and/or backbone movements upon ligand docking. The results were optimized using the best predictions made by the 20 ns molecular dynamics.

### Quantum similarity analysis

*Molecular quantum similarity measure*

A molecular quantum similarity measure (MQSM) amid two A and B systems, known as Z _AB_, compares two molecules using their respective density functions (DFs). Both DFs can be multiplied and integrated in terms of their electronic coordinates, which are then weighted using a predetermined positive operator Ω(r _1_,r _2_): ^11^^–^^13^

$$Z_{\mathrm{AB}}=\left\langle \rho_{A}\left|\Omega \right|\rho_{B}\right\rangle =\int \int \rho_{A}\left(r_{1}\right)\Omega \left(r_{1},r_{2}\right)\rho_{B}\left(r_{2}\right){\mathrm{dr}}_{1}{\mathrm{dr}}_{2}$$ (1)

The nature of the operator used in Equation 1 provides the information being compared between the two systems and determines the similarity measure. For instance, if the chosen operator is the Dirac delta function (an efficient approach for functions with high peak values, such as the electronic density), *i.e.*, Ω(r _1_,r _2_)= δ(r _1_- r _2_). One of the first similarity metrics employed is the overlapping MQSM; another widely used alternative is the Coulomb operator, *i.e.*, Ω(r _1_,r _2_)=|‌ _1_- r _2_|‌‌ ^-1^, resulting in a Coulombic MQSM. Even if the two molecules are equivalent, a similarity measure can be employed, which is known as a self-similarity measure (Z _AA_ for the case of molecule A). ^12^

Given a group of N molecules, we can generate a similarity measure for each of them with regard to the other molecules in the group, including themselves. With this, a symmetric matrix can be constructed where the i-th column of the matrix is the collection of all similarity measures between the i-th molecule and each of the components of the group, including itself. Each vector (column of the matrix) is a discrete representation (in N dimensions) of the i-th structure. These sets of vectors are a set of chemical descriptors that do not simply express another set of molecular descriptors as is often done, but rather each descriptor has its own set of unique properties. ^12^^,^^13^

i. It is universal in that it can be derived from any collection of molecules and any individual molecule within that group.
ii. It is impartial because there are no other possibilities available in the construction process than those given the density functions and similarity measurements involved.

*MQSM similarity index manipulation and visualization techniques*

The similarity measure obtained for the group is unique once we have chosen a group of study objects and the operator in Equation 1; however, these measures can be combined or transformed to gain a new class of additional terms, which can be called Quantum Similarity Indices (QSI).

There are varieties of possible manipulations of the MQSM that result in a variety of QSI definitions. The most common ones are the following and are used in this paper ^13^^–^^16^:

*Carbó's similarity index*

Carbó's similarity index between two molecules I and J is constructed from Equation 2. Because this index is also known as the cosine similarity index, it corresponds to the cosine of the angle formed by the density functions when considered as vectors. For any pair of compared molecules, this Carbo QSI has a value between 0 and 1, depending on the similarity between the two molecules (when I = J, the index approaches 1). ^13^^–^^17^

$$C_{\mathrm{IJ}}\left(\Omega \right)=z_{\mathrm{IJ}}\left(\Omega \right){\left[z_{\mathrm{II}}\left(\Omega \right)z_{\mathrm{JJ}}\left(\Omega \right)\right]}^{-1/2}$$ (2)

*Quantum similarity using Euclidean distance*

Taking into account the similarity of Equation 3:

$$D_{\mathrm{IJ}}\left(k,x,\Omega \right)={\left[k\left(z_{\mathrm{II}}\left(\Omega \right)+z_{\mathrm{JJ}}\left(\Omega \right)\right)/2-{\mathrm{xz}}_{\mathrm{IJ}}\left(\Omega \right)\right]}^{1/2},x\left[0,k\right]$$ (3)

It can be simplified to the so-called Euclidean distance index when *k* = *x* = 2 and can also be defined as follows:

$$D_{\mathrm{IJ}}\left(\infty ,\Omega \right)=\max\left(z_{\mathrm{II}}\left(\Omega \right),z_{\mathrm{JJ}}\left(\Omega \right)\right)$$ (4)

This Equation 4 forms the distance index of infinite order. ^18^

*MQSM definition*

Quantum similarity measurements are in accordance with psychological perception and the apparent principle of similarity: “the more similar two molecules, the more similar their properties are.” This statement necessitates the construction of a molecule-to-molecule comparison, and the comparison of their densities is used to derive a quantitative measure of the quantum similarity of two molecules. Generally, the MQSM can be defined as the integral computational measure between two density functions, where the density functions are multiplied and integrated for the electronic coordinates in the relevant domain. ^19^^–^^21^

$$Z_{\mathrm{IJ}}\left(\Omega \right)=\left\langle \rho_{I}\left|\Omega \right|\rho_{J}\right\rangle =\int \int \rho_{I}\left(r\right)\Omega \left(r_{1},r_{2}\right)\rho_{J}\left(r_{2}\right){\mathrm{dr}}_{1}{\mathrm{dr}}_{2}\in R^{+}$$ (5)

{r _1_, r _2_} are sets of electronic coordinates related to the corresponding wave function, and the operator Ω (r _1_,r _2_) is positively defined and supported on the electronic coordinates. ^22^^–^^26^

*Types of measurements in molecular quantum similarity*

The types of measurements are mainly determined by the information required, the selection of supported operators and the form of MSQM. ^18^^–^^20^

MQSM overlap considering Equation 2:

The distribution of Dirac's delta, Ω (r1, r2) = δ (r1, r2), is the most typical and intuitive choice for such a positively defined operator. These selections transform the broad definition of MQSM to compute the overlap MQSM that obtains measurements of the volume by both electronic density functions when they are superimposed. ^17^^–^^20^

The Dirac delta distribution, Ω (r1, r2) = δ (r1, r2), is the most typical and intuitive choice for a positive definite operator. This choice transforms the broad definition of MQSM to compute the overlap MQSM. ^17^^–^^20^ The MQSM calculates the degree of overlap between molecular densities using information about the electron “concentration” in the molecule ^16^^–^^21^:

$$Z_{\mathrm{IJ}}\left(\Omega \right)=\int \int \rho_{I}\left(r_{1}\right)\delta \left(r_{1}-r_{2}\right)\rho_{J}\left(r_{2}\right){\mathrm{dr}}_{1}{\mathrm{dr}}_{2}=\int \rho_{I}\left(r\right)\rho_{J}\left(r\right)\mathrm{dr}$$ (6)

MQS coulomb considering Equation 2:

When the operator (Ω) is replaced with the Coulomb operator, $\Omega \;\left(r_{1},r_{2}\right)=\frac{1}{\left|r_{1}-r_{2}\right|}$ , the coulomb MQS is generated, which defines the electrostatic Coulomb repulsion energy between two charge densities ^20^^,^^21^:

$$Z_{\mathrm{IJ}}\left(\Omega \right)=\int \int \rho_{I}\left(r_{1}\right)\frac{1}{r_{1}-r_{2}}\rho_{J}\left(r_{2}\right){\mathrm{dr}}_{1}{\mathrm{dr}}_{2}$$ (7)

The Coulomb operator affects the overlap density functions. When considering molecular density functions as an electron distribution in space, this equation is simply an extension of the Coulomb operator for a distribution of continuous charge, thus can be used as electrostatic potential descriptors in some instances. This operator is correlated to electrostatic interactions and obtains the measurement of electrostatic repulsion between electronic distributions. ^15^^–^^19^

Euclidean distance index considering Equation 3:

Another major transformation that can be expressed in terms of the classical distance is:

dab=∑j=1pΔxjk1k (8)

Here $\Delta x_{j}=x_{\mathrm{aj}}-x_{\mathrm{bj}}$ is the distance between *a* and *b*, and *k* = 2 is the definition of distance. Subsequently, the Euclidean distance between A and B (two quantum objects) is defined by: ^17^^–^^21^

$$d_{\mathrm{ab}}=\sqrt{{\left(x_{a}-x_{b}\right)}^{2}}.$$ (9)

Occasionally it is written as: $D_{\mathrm{AB}}=\sqrt{Z_{\mathrm{AA}}+Z_{\mathrm{BB}}+Z_{\mathrm{AB}}}$ , where *D*_AB_ has values in the range of [0,∞} but for situations where the compared items are identical, it converges to zero ^17^^–^^21^:

$$D_{\mathrm{AB}}=0$$ (10)

The norm of the differences of the density functions can be used in the definition Equation 9. The distance or dissimilarity index can be used to define the Euclidean distance index, which can also be represented as ^15^^–^^21^:

$$D_{\mathrm{AB}}=\left\|\rho_{A}-\rho_{B}\right\|=\sqrt{{\left(\rho_{A}-\rho_{B}\right)}^{2}}$$ (11)

*Alignment method: Topo-Geometrical Superposition Algorithm (TGSA)*

In this research, the TGSA ^23^ approach has been used to align the data. Gironés proposed the TGSA, in addition to programming and implementing it. This method assumes that the best way to align molecules is to superimpose them on a typical skeleton, with only the type atoms and the interatomic bonding interactions. The program accomplishes its purpose by examining the pairs of molecules and aligning the common substructure for a group. ^23^ Only topological and geometrical considerations are used in the procedure, including molecular topology and how distant bonds are compared. The superposition of two molecules is unique and is independent of the type of operator used to determine the similarity. ^23^^,^^24^

### Chemical reactivity analysis

Our research group has used several reactivity indices with Quantum Similarity. ^26^^–^^35^ The reactivity indices used in this work are chemical potential (μ), ^36^^–^^38^ hardness (ɳ), ^39^ and electrophilicity (ω), ^38^^,^^39^ which will be calculated.

$$\mu \approx \frac{E_{\text{LUMO}}+E_{\text{HOMO}}}{2}$$ (12)

$$\eta \approx E_{\text{LUMO}}-E_{\text{HOMO}}$$ (13)

The electrophilicity index (ω) measures the stabilization energy of the system when it is saturated by electrons from the external environment, and is mathematically defined as ^38^^,^^39^:

$$\omega =\frac{\mu^{2}}{2\eta }$$ (14)

In this study, the local reactivity descriptors are the Fukui functions. Equations 15 and 16 represent the response of the chemical potential of a system to changes in the external potential. It is defined as the derivative of the electronic density with regard to the number of electrons at the constant external potential:

$$f_{k}^{+}\approx \int_{k}\left[\rho_{N+1}\left(\vec{r}\right)-\rho_{N}\left(\vec{r}\right)\right]=\left[q_{k}\left(N+1\right)-q_{k}\left(N\right)\right]$$ (15)

$$f_{k}^{-}\approx \int_{k}\left[\rho_{N}\left(\vec{r}\right)-\rho_{N-1}\left(\vec{r}\right)\right]=\left[q_{k}\left(N\right)-q_{k}\left(N-1\right)\right]$$ (16)

Where $\left(f_{k}^{-}\right)$ is for nucleophilic attack and $\left(f_{k}^{-}\right)$ for the electrophilic attack. ^40^^–^^42^ In this approach, using the global and local reactivity descriptors makes it possible to compare the molecular reactivity at the sample set. All the structures were developed using **M02X/6–31G(d, p)** ^43^ methods in **Gaussian 09** package. ^44^

## Results

### Docking results

**Figure 1.**
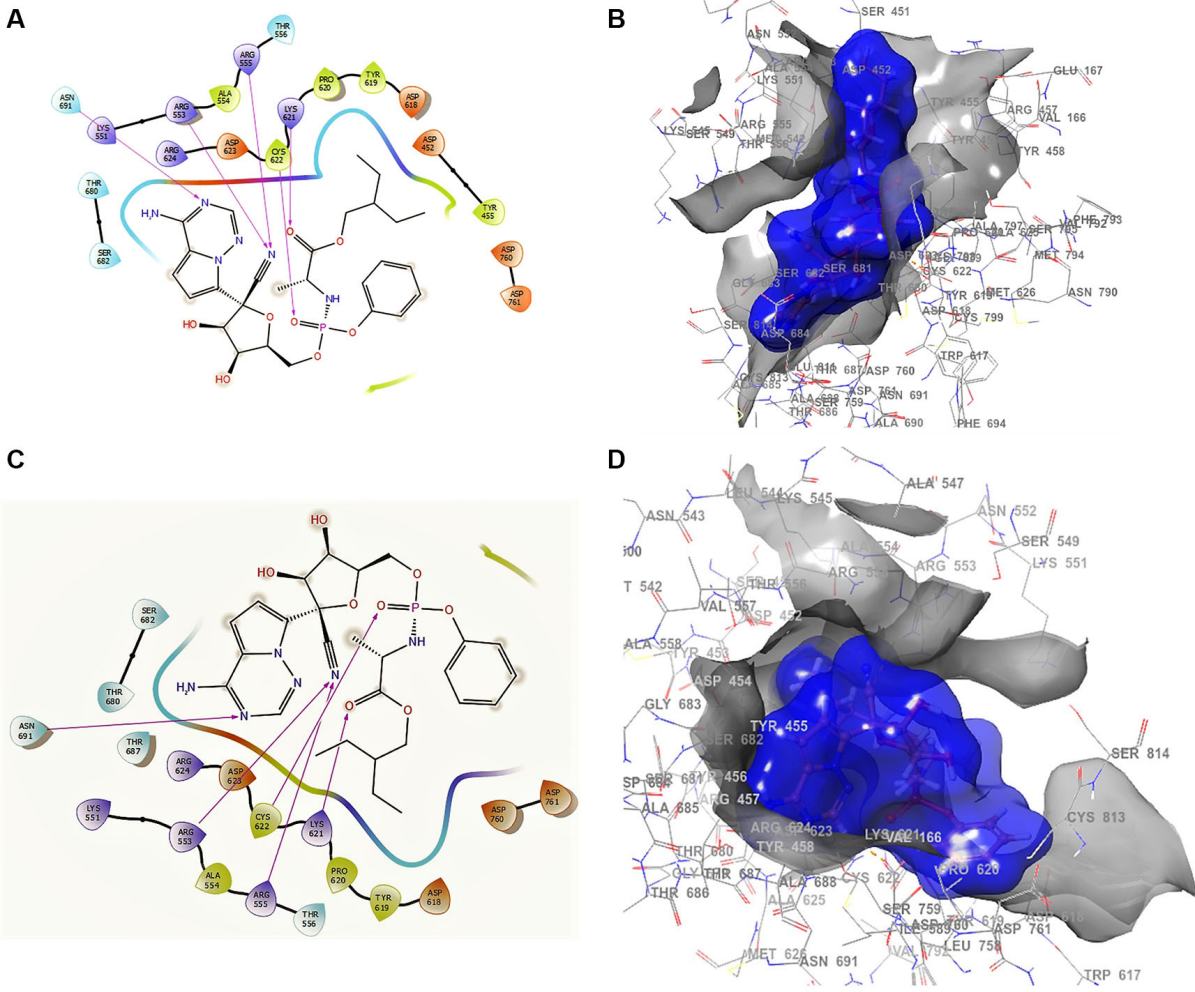
Docking outcomes using remdesivir. A. Docking interactions using remdesivir with the crystal structure of SARS-CoV-2 RdRp (PDB accession number 6M71). B. Docking interactions using Receptor (gray) and ligand (blue) surfaces. C. Docking interactions with the receptor site. D. Surface of the binding pocket with the receptor site. SARS-CoV-2, severe acute respiratory syndrome coronavirus 2; RdRp, RNA-dependent RNA polymerase; PDB, Protein Data Bank.

In this work, the stabilization process of a set of ligands related with activity against the novel SARS-CoV-2 was studied to understand the main ligand interactions in the active site, starting from the PDB crystal structure of SARS-CoV-2-dependent RNA polymerase code: 6M71. ^45^^,^^46^ The crystal structure of SARS-CoV-2 RdRp was selected taking into account the outcomes reported by Elfiky *et al.* ^47^ Figure 1 ^51^ shows the docking interactions using remdesivir. In the conformation of Figure 1A and B, the docking interaction for the structure of remdesivir with a higher RMSD shows an -H bond with ARG555, ARG553 with two lengths of 2.28 Å and a length of 2.35 Å, LYS621 with a length of 2.22 Å, CYS622 with a length of 2.42 Å, and ASN691 with a length of 2.38 Å.

Another of the best conformations of remdesivir ( Figure 1C and D) involved a -H bond with residue ASN691 with a length of 2.46Å, two ARG553 and ARG555 with lengths of 2.35Å and 2.38Å, CYS622 with a length of 2.41Å, and LYS621 with a length of 2.39Å. The interactions with the residue ARG553 and ARG555 and LYS621 were very similar to Figure 1A and B. These interactions play an important role in the interaction of the active site, generating a good bonding surface (see Figure 1B and D).

**Figure 2.**
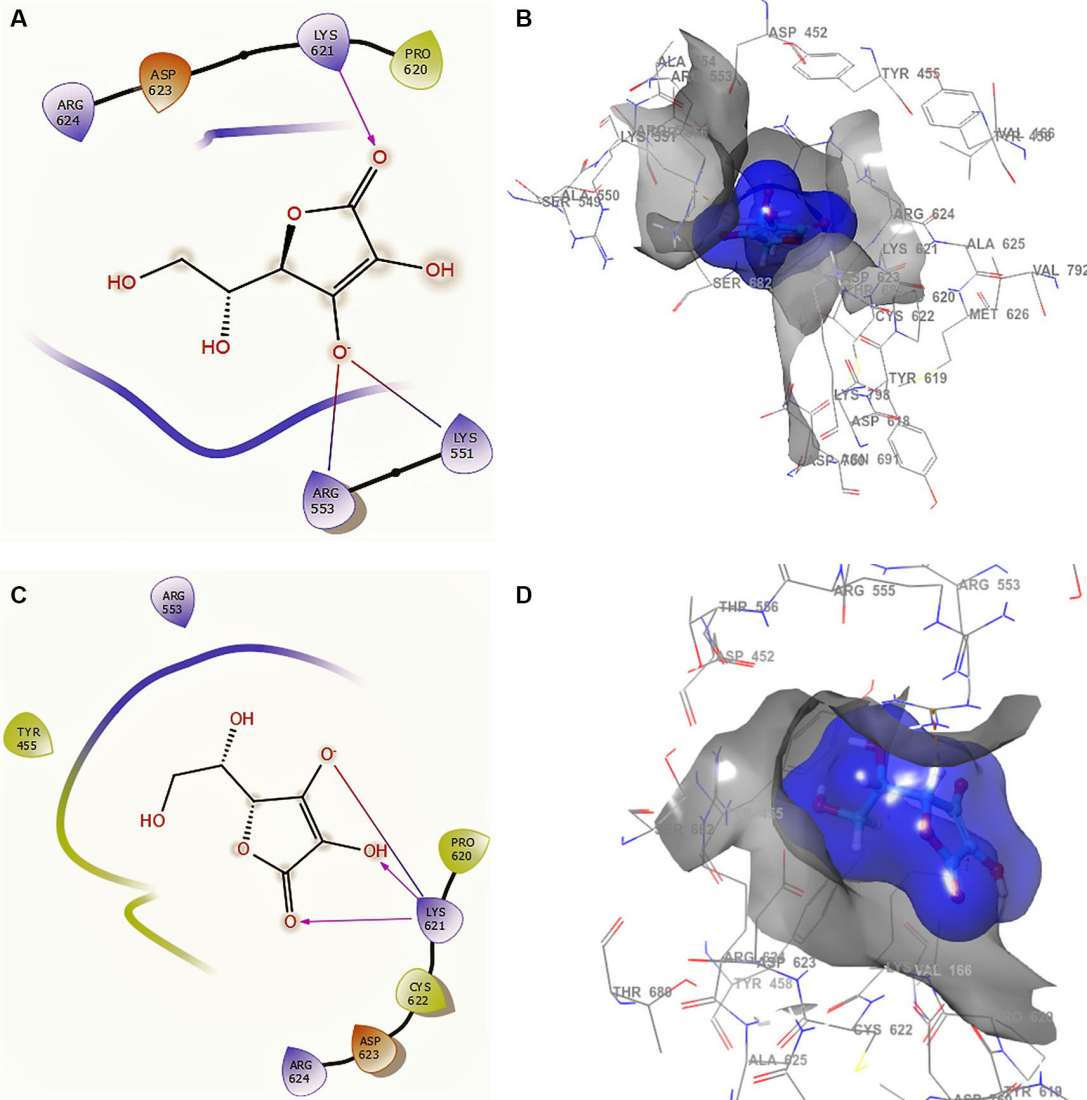
Docking outcomes using ascorbic acid vitamin C. A. Docking interactions using ascorbic acid vitamin C with the crystal structure of SARS-CoV-2 RdRp (PDB accession number 6M71). B. Receptor (gray) and ligand (blue) surfaces. C. Docking interactions with the receptor site. D. Surface of the binding pocket with the receptor site. SARS-CoV-2, severe acute respiratory syndrome coronavirus 2; RdRp, RNA-dependent RNA polymerase; PDB, Protein Data Bank.

Figure 2 shows the docking interactions using ascorbic acid vitamin C structure of SARS-CoV-2 RdRp from SARS-CoV-2. The ligand and receptor surfaces show the active site zone defined by the stabilization interactions. A phosphate group was found to reside well inside a local binding pocket within the grip with residue CYS622.

In Figure 2A and B, the ascorbic acid vitamin C has -H bonds with the residues LYS621 at a distance of 2.20Å, ARG553 at a distance of 1.86Å, and LYS551 at a distance of 1.86Å, respectively. The ligand has the same interaction as remdesivir with residue LYS621. This conformation showed a good stabilization in the pocket, generating a good contour surface (see blue surface). Conformation 2 ( Figure 2C and D) shows three -H bonds with the same residue LYS621 at distances 2.18Å, 2.25Å and 2.27Å. This residue LYS621 is also shown in the conformations of remdesivir.

**Figure 3.**
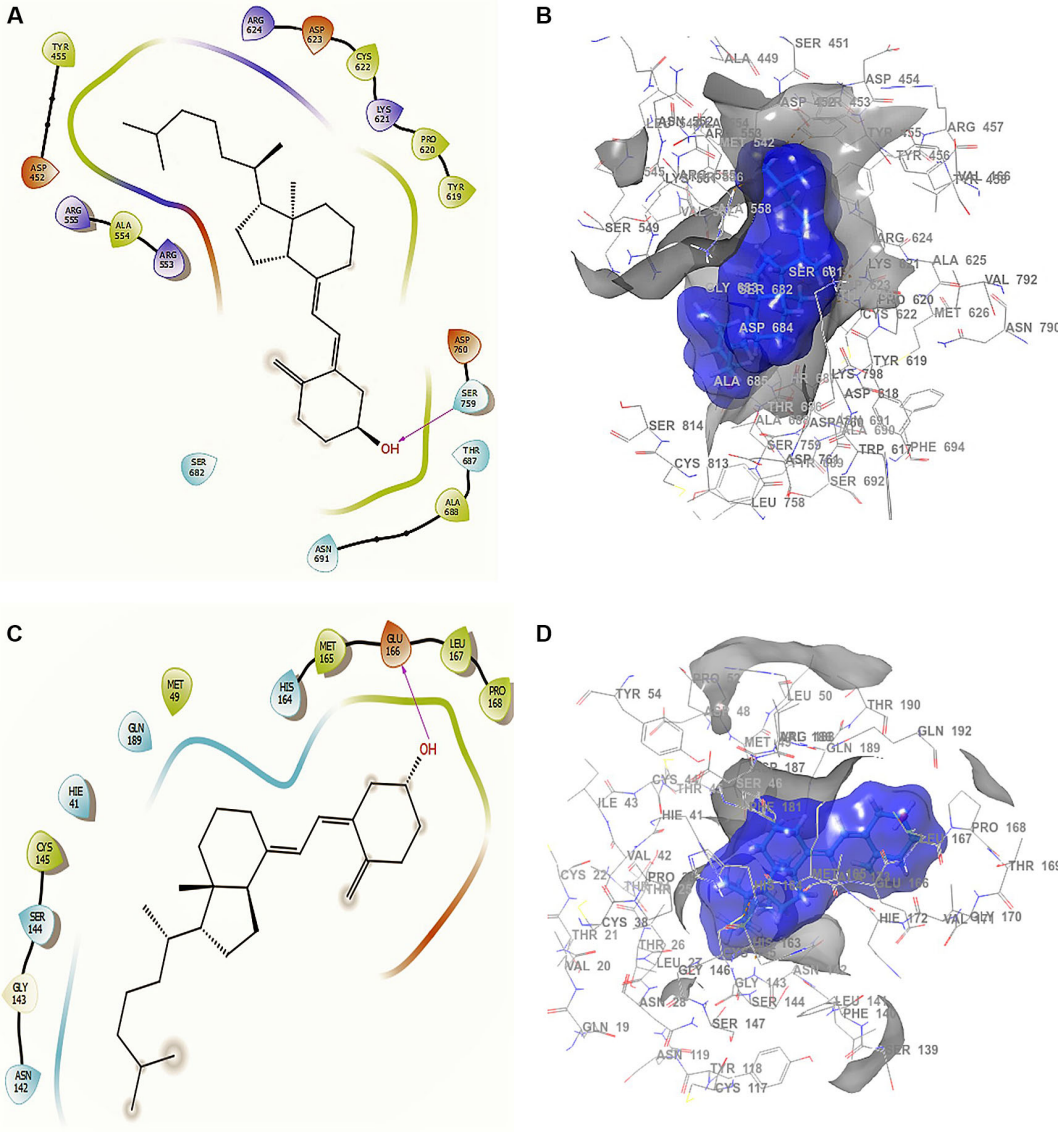
Docking outcomes using cholecalciferol vitamin D. A. Docking interactions of cholecalciferol vitamin D with the crystal structure of SARS-CoV-2 RdRp (PDB accession number 6M71). B. Receptor (gray) and ligand (blue) surfaces. C. Docking interactions with the receptor site. D. Surface of the binding pocket with the receptor site. SARS-CoV-2, severe acute respiratory syndrome coronavirus 2; RdRp, RNA-dependent RNA polymerase; PDB, Protein Data Bank.

Conformation 1 of Figure 3A and B shows the structure of cholecalciferol vitamin D, the largest RMSD corresponding to an -H bond with the residue SER759 at a length of 2.24Å. For conformation 2 in Figure 3C and D the -H bond is with the residue GLU166 with a length of 2.28Å. Finally, the compound has interactions with the residue TRP617 with a length 2.26 Å. Although this compound only interacted with the residue SER759, the ligand surface is well defined near the receptor, even though it is a single -H bond. The interaction is good enough to generate an active conformation with stabilizing capacity ( Figure 3C and D), in agreement with previous reported results. ^48^

**Figure 4.**
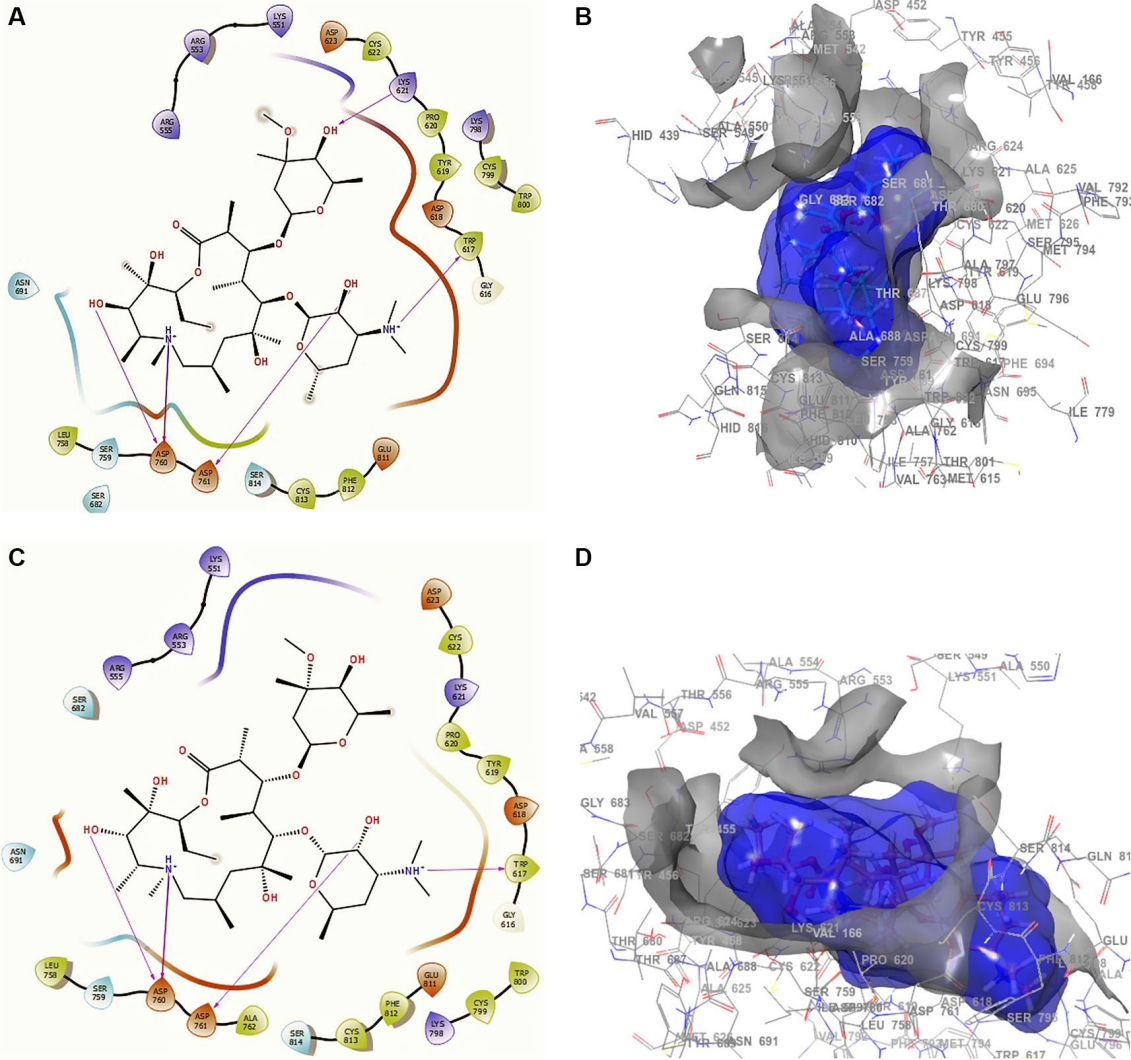
Docking outcomes using azithromycin. A. Docking interactions using azithromycin with the crystal structure of SARS-CoV-2 (PDB accession number 6M71). B. Receptor (gray) and ligand (blue) surfaces with a receptor (gray) and ligand (blue) surfaces. C. Docking interactions with the receptor site. D. Surface of the binding pocket with the receptor site. SARS-CoV-2, severe acute respiratory syndrome coronavirus 2; RdRp, RNA-dependent RNA polymerase; PDB, Protein Data Bank.

Conformation 1 of azithromycin ( Figure 4A and B), like those of remdesivir and ascorbic acid vitamin C, has -H bond with LYS621 with a length of 2.28Å. In addition, it has two interactions with the residue ASP760 at lengths of 2.26Å and 2.34Å, with ASP761 at a length of 2.32Å. On the other hand, for conformation 2 of azithromycin of Figure 4C and D, we can see two -H bonds with the residues ASP760 with lengths of 2.41Å and 2.38Å, ASP761 with a length of 2.38Å and TRP617 with a length of 2.28Å. These ASP760, ASP761 and TRP617 interactions agree with the recent work published by Abdrabbo *et al.* ^49^ In this work, once the stable binding mode was located, free energy perturbation (FEP) calculations were carried out to estimate the binding affinity of RemTP and ATP to COVID-19, and to identify the key residues in the binding process.

**Figure 5.**
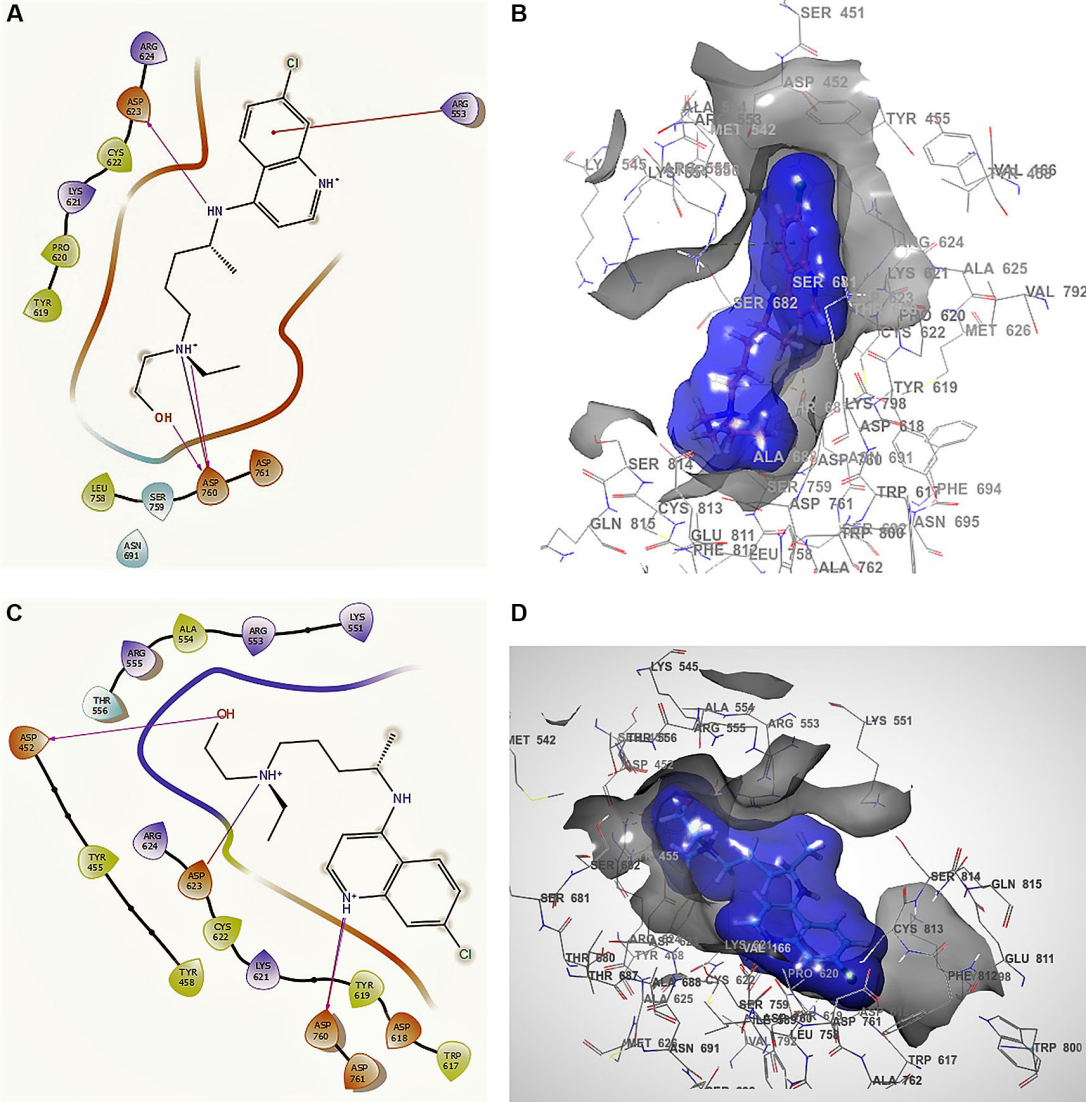
Docking outcomes using hydroxychloroquine. A. Docking interactions using hydroxychloroquine with the crystal structure of SARS-CoV-2 RdRp (PDB accession number 6M71). B. Receptor (gray) and ligand (blue) surfaces with a receptor (gray) and ligand (blue) surfaces. C. Docking interactions with the receptor site. D. Surface of the binding pocket with the receptor site. SARS-CoV-2, severe acute respiratory syndrome coronavirus 2; RdRp, RNA-dependent RNA polymerase; PDB, Protein Data Bank.

The docking interactions in conformation 1 of Figure 5A and B for hydroxychloroquine with higher RMSD shows a -H bond with the residue ARG553 with a length of 2.38Å, with ASP760 at lengths of 2.36 Å, 2.28 Å and 2.32 Å. For the best conformation 2, Figure 5C and D involved ASP462, ASP623 and ASP760 with lengths of 2.36 Å, 2.31 Å and 2.28 Å, respectively.

In this study, a molecular quantum similarity study was developed to systematically investigate and analyze the structural and electronic considerations involved in active site stabilization. An electronic similarity study has been designed to find the relationship between ligands and the active site of RdRp proteins.

### Molecular quantum similarity indices

Because conformations are crucial in docking studies, we have analyzed the impact that molecular alignment has on the generation of interactions (-H bond) at the active site. These molecular alignments, which generated the best poses, were related to potential COVID-19 inhibitors. For this analysis we have used four similarity descriptors (structural, electronic and their Euclidean distances) and molecular quantum similarity indices were calculated using the overlap and coulomb descriptors ( Equations 6 and 7).

The compounds analyzed in the docking results were compared with a series of ligands with SARS-CoV-2 activity such as abacavir, acyclovir, amprenavir, ascorbic acid vitamin C, azithromycin, baloxavir, boceprevir, cholecalciferol vitamin D, cidofovir, edoxudine, emtricitabine, hydroxychloroquine and remdesivir. These compounds have been associated with COVID-19 treatment.

The molecular quantum similarity indices are defined on the interval (0,1), where **0** means dis-similarity and **1** means self-similarity. Table 1 shows the molecular quantum similarity indices using the overlap descriptor. Based on the distribution of Dirac's delta, Ω (r1, r2) = δ (r1, r2), this operator can be related to the structural characteristics of the compound analyzed. The highest values were for abacavir/acyclovir (0.6286) with an Euclidean distance of 3.5612 ( Table 2), emtricitabine/abacavir (0.6011) with an Euclidean distance of 3.7645 and emtricitabine/edoxudine (0.6079) with a Euclidean distance of 3.8020. The lowest values were for boceprevir/azithromycin (0.1374), and remdesivir/cholecalciferol (0.1476) with a Euclidean distance of 7.1944.

**Table 1.** Molecular quantum similarity indices using overlap descriptor ( Equation 7).

O_Hab	Abaca	Acyc	Ampre	Asco	Azythr	Balox	Boce	Chole	Cido	Edox	Emitri	Hidrox	Remd
**Abaca**	**1.0000**												
**Acyc**	0.6286	**1.0000**											
**Ampre**	0.3022	0.2988	**1.0000**										
**Asco**	0.3893	0.5072	0.3341	**1.0000**									
**Azythr**	0.1858	0.2578	0.1374	0.2185	**1.0000**								
**Balox**	0.2715	0.2966	0.3104	0.2532	0.2008	**1.0000**							
**Boce**	0.2478	0.2867	0.2088	0.2891	0.1374	0.2577	**1.0000**						
**Chole**	0.2212	0.1949	0.2088	0.2727	0.2995	0.2675	0.2497	**1.0000**					
**Cido**	0.4560	0.4153	0.3620	0.3150	0.2857	0.3713	0.2736	0.2260	**1.0000**				
**Edox**	0.4402	0.5878	0.2614	0.4179	0.2250	0.3781	0.2715	0.2459	0.5160	**1.0000**			
**Emitri**	0.6011	0.4913	0.2744	0.3292	0.2009	0.4844	0.2535	0.3182	0.5648	0.6079	**1.0000**		
**Hidrox**	0.4048	0.3762	0.2833	0.3034	0.2483	0.3419	0.3054	0.3164	0.3971	0.2726	0.4658	**1.0000**	
**Remd**	0.4527	0.3865	0.2473	0.3471	0.3027	0.2359	0.2694	0.1476	0.3607	0.2540	0.3813	0.3339	**1.0000**

O_Hab, overlap index; remd, remdesivir; abaca, abacavir; acyc, acyclovir; ampre, amprenavir; asco, ascorbic acid vitamin C; azythr, azithromycin; balox, baloxavir; boce, boceprevir; chole, cholecalciferol vitamin D; cido, cidofovir; edox, edoxudine; emitri, emtricitabine; hidrox, hydroxychloroquine.

**Table 2.** Euclidean molecular quantum similarity distance using overlap descriptor ( Equation 11).

O_Dab	Abaca	Acyc	Ampre	Asco	Azythr	Balox	Boce	Chole	Cido	Edox	Emitri	Hidrox	Remd
**Abaca**	**0.0000**												
**Acyc**	3.5612	**0.0000**											
**Ampre**	5.9345	5.8864	**0.0000**										
**Asco**	4.4872	3.9524	5.6923	**0.0000**									
**Azythr**	7.6457	7.3080	8.5294	7.4150	**0.0000**								
**Balox**	6.5703	6.4155	7.0473	6.5320	8.5827	**0.0000**							
**Boce**	6.2649	6.0508	7.2027	5.9841	8.6147	7.3953	**0.0000**						
**Chole**	5.4578	5.4606	6.4615	5.1121	7.2809	6.7187	6.4090	**0.0000**					
**Cido**	4.5577	4.6524	5.8175	4.9550	7.3381	6.2563	6.3043	5.6314	**0.0000**				
**Edox**	4.5316	3.8283	6.1662	4.4733	7.5427	6.1713	6.2393	5.4610	4.3697	**0.0000**			
**Emitri**	3.7645	4.1778	6.0541	4.7147	7.5931	5.6486	6.2518	5.1193	4.0875	3.8020	**0.0000**		
**Hidrox**	4.6240	4.6534	6.0396	4.8340	7.4159	6.3017	6.0641	5.1517	4.8313	5.2048	4.3916	**0.0000**	
**Remd**	5.7747	6.0282	7.3447	6.1492	8.0120	7.7967	7.3225	7.1944	6.2877	6.6851	6.0992	6.3185	**0.0000**

O_Dab, overlap Index_Distance; remd, remdesivir; abaca, abacavir; acyc, acyclovir; ampre, amprenavir; asco, ascorbic acid vitamin C; azythr, azithromycin; balox, baloxavir; boce, boceprevir; chole, cholecalciferol vitamin D; cido, cidofovir; edox, edoxudine; emitri, emtricitabine; hidrox, hydroxychloroquine.

The molecular quantum similarity indices using the Coulomb descriptor were calculated to obtain insights into the electronic similarity (see Table 3). The Coulomb descriptor is related to the electronic similarity in the molecular set. Table 3 shows that the highest values for emtricitabine/edoxudine (0.9390) with an Euclidean distance of 14.4795 (see Table 4), emtricitabine/abacavir (0.9311) with an Euclidean distance of (16.2459) and edoxudine/acyclovir (0.9165) with a Euclidean distance of 22.2556. Unlike the overlap descriptor, the values are near to 1. This means that despite having significant structural differences, these structures have high electronic similarities.

**Table 3.** Molecular quantum similarity indices using coulomb descriptor ( Equation 7).

C_Hab	Abaca	Acyc	Ampre	Asco	Azythr	Balox	Boce	Chole	Cido	Edox	Emitri	Hidrox	Remd
**Abaca**	**1.0000**												
**Acyc**	0.9034	**1.0000**											
**Ampre**	0.7589	0.6938	**1.0000**										
**Asco**	0.7692	0.8584	0.8159	**1.0000**									
**Azythr**	0.7987	0.7591	0.7366	0.7690	**1.0000**								
**Balox**	0.7712	0.8035	0.8581	0.8180	0.7438	**1.0000**							
**Boce**	0.8198	0.7327	0.8100	0.8051	0.6053	0.8846	**1.0000**						
**Chole**	0.7745	0.6049	0.8061	0.5801	0.8534	0.8325	0.8294	**1.0000**					
**Cido**	0.8396	0.7541	0.8373	0.8048	0.8545	0.8779	0.8335	0.8043	**1.0000**				
**Edox**	0.8804	0.9165	0.6690	0.8313	0.8003	0.8685	0.7723	0.7596	0.9065	**1.0000**			
**Emitri**	0.9311	0.8270	0.7762	0.8068	0.7928	0.8095	0.8290	0.8096	0.8889	0.9390	**1.0000**		
**Hidrox**	0.7981	0.8487	0.8119	0.8494	0.8438	0.8494	0.8220	0.8301	0.8934	0.7461	0.8422	**1.0000**	
**Remd**	0.8169	0.8007	0.8460	0.7375	0.8818	0.8189	0.7865	0.6615	0.8723	0.7036	0.8177	0.8242	**1.0000**

C_Hab, Coulomb; remd, remdesivir; abaca, abacavir; acyc, acyclovir; ampre, amprenavir; asco, ascorbic acid vitamin C; azythr, azithromycin; balox, baloxavir; boce, boceprevir; chole, cholecalciferol vitamin D; cido, cidofovir; edox, edoxudine; emitri, emtricitabine; hidrox, hydroxychloroquine.

**Table 4.** Euclidean molecular quantum similarity distance using coulomb descriptor ( Equation 11).

C_Dab	Abaca	Acyc	Ampre	Asco	Azythr	Balox	Boce	Chole	Cido	Edox	Emitri	Hidrox	Remd
**Abaca**	**0.0000**												
**Acyc**	19.0791	**0.0000**											
**Ampre**	45.1211	50.2246	**0.0000**										
**Asco**	28.1964	18.9977	46.0616	**0.0000**									
**Azythr**	72.2227	78.0583	69.5925	80.4297	**0.0000**								
**Balox**	50.0532	50.7112	38.8947	52.6116	68.4096	**0.0000**							
**Boce**	44.3478	51.9602	43.5992	50.5473	82.1636	35.6613	**0.0000**						
**Chole**	35.5204	44.8126	40.4112	45.7335	61.8020	42.3094	40.7413	**0.0000**					
**Cido**	24.6924	28.6770	39.8670	25.7443	69.2265	42.8673	43.6975	33.4262	**0.0000**				
**Edox**	21.1305	16.8664	50.8603	23.5396	73.1987	44.1587	48.1913	36.5204	18.4362	**0.0000**			
**Emitri**	16.2459	22.2556	45.4051	22.7597	75.5667	49.6984	46.0082	33.6813	19.7500	14.4795	**0.0000**		
**Hidrox**	30.4613	27.0732	40.1966	28.5071	65.8107	42.1731	42.4122	31.5000	22.5763	33.6859	27.2762	**0.0000**	
**Remd**	49.7443	53.8676	42.0152	59.2508	49.6235	46.4932	49.7802	59.1914	46.2112	57.5336	52.2004	46.9765	**0.0000**

C_Dab, Coulomb_Distance; remd, remdesivir; abaca, abacavir; acyc, acyclovir; ampre, amprenavir; asco, ascorbic acid vitamin C; azythr, azithromycin; balox, baloxavir; boce, boceprevir; chole, cholecalciferol vitamin D; cido, cidofovir; edox, edoxudine; emitri, emtricitabine; hidrox, hydroxychloroquine.

### Global and local reactivity indices

This work has also explored the global and local chemical reactivity indices within the DFT framework. Table 5 shows that the least reactive molecule was hydroxychloroquine, whose electronic chemical potential μ= -1.7070 eV, chemical hardness Ƞ=1.606 eV, softness *S*=0.6226 eV and electrophilicity ω=0.4536 eV. The most reactive compound was baloxavir-marboxil hydroxychloroquine, whose electronic chemical potential μ= -3.7335 eV, chemical hardness Ƞ=2.1441 eV, softness *S*=0.4664 eV and electrophilicity ω=1.6253 eV. These electrophilicity values play an essential role in ligands stabilized by non-covalent interactions and determine the stability of the active site.

**Table 5.** Global chemical reactivity indices.

Compound	Chemical potential (μ), eV	Chemical hardness (Ƞ), eV	Softness (S), eV	Electrophilicity (ω), eV
Abacavir	-2.6330	2.4193	0.4133	0.7164
Acyclovir	-3.1320	2.5638	0.3900	0.9565
Amprenavir	-3.4399	2.5898	0.3861	1.1423
Ascorbic acid vitamin C	-3.4399	2.5898	0.3861	1.1423
Azithromycin	-3.5621	2.5532	0.3916	1.2136
Baloxavir-marboxil	-3.7335	2.1441	0.4664	1.6253
Cholecalciferol vitamin D	-2.9825	2.4249	0.4123	0.9171
Cidofovir	-3.4533	2.5583	0.3908	1.1653
Edoxudine	-3.6300	2.6202	0.3816	1.2572
Hydroxychloroquine	-1.7070	1.606	0.6226	0.4536
Remdesivir	-3.6097	2.3253	0.4300	1.4009

**Figure 6.**
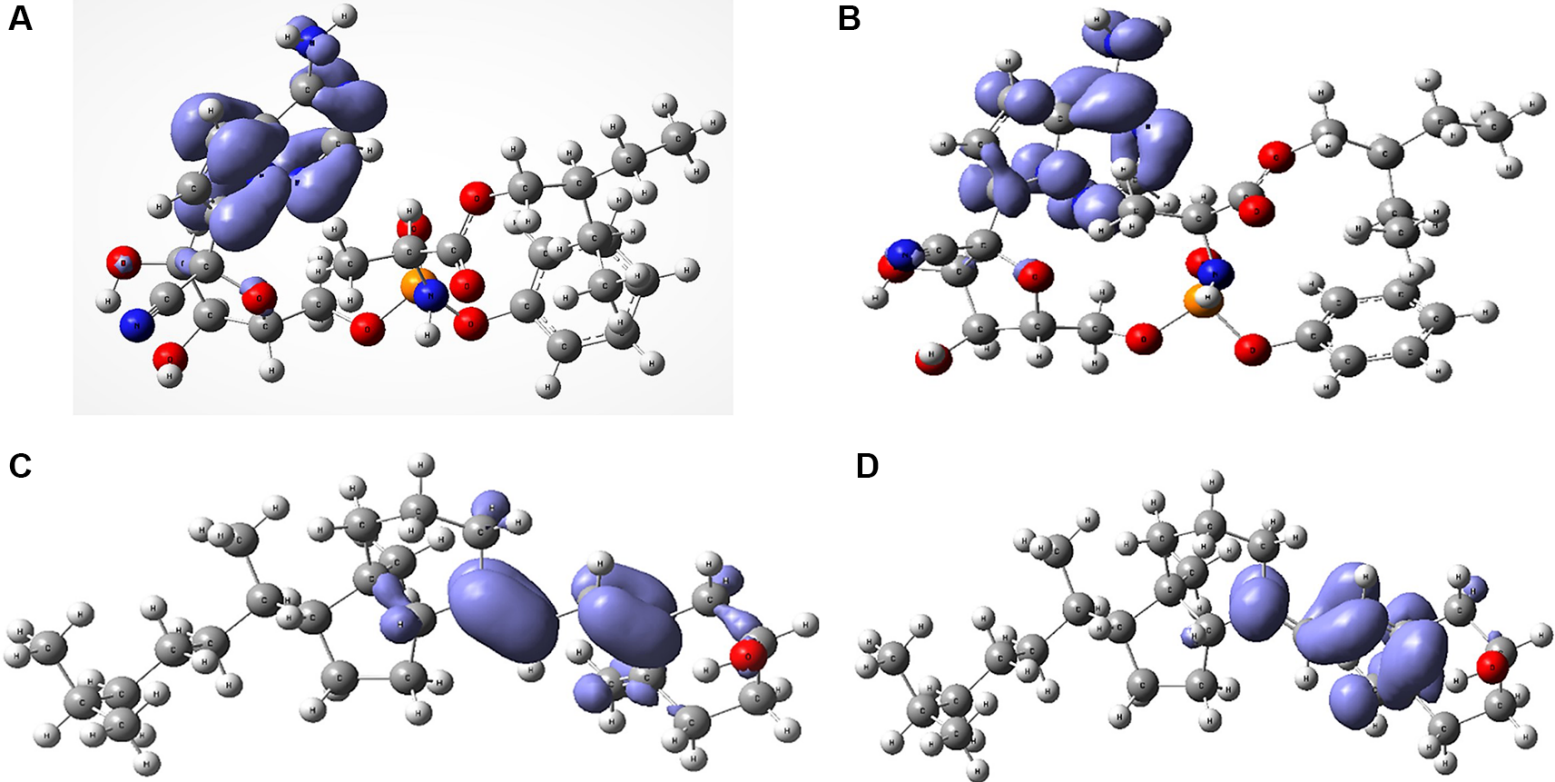
Local chemistry reactivity indices. A. Fukui function $f^{-}\left(r\right)\approx {\left|\text{HOMO}\right|}^{2}$ and B. Fukui Function $f^{+}\left(r\right)\approx {\left|\text{HOMO}\right|}^{2}$ related with remdesivir. C. Fukui Function $f^{-}\left(r\right)\approx {\left|\text{HOMO}\right|}^{2}$ and D. $f^{+}\left(r\right)\approx {\left|\text{HOMO}\right|}^{2}$ related with cholecalciferol vitamin D.

The compounds with the highest chemical potential (negative electronegativity) were hydroxychloroquine with μ= -1.7070 eV, abacavir with μ= -2.6330 eV and cholecalciferol vitamin D with μ= -2.9825 eV. These compounds are commonly used in COVID-19 treatment. ^48^^–^^50^ On the other hand, abacavir is used to treat HIV infection and some studies have shown its relationship with COVID-19. ^50^ However, some countries are skeptical about its use for COVID-19 treatment. Other important compounds often used for the treatment of SARS-CoV-2 virus are azithromycin and remdesivir, with a chemical reactivity of μ= -3.5621 eV and -3.6097 eV, chemical hardness Ƞ=2.5532 eV and 2.3253 eV, softness *S*=1.2136 eV and 0.4300 eV and electrophilicity ω=1.6253 eV and 1.4009 eV, respectively. Both are very reactive and have high electrophilicity values. Figure 6 shows the Fukui functions that have been used to describe the local chemical reactivity of remdesivir and cholecalciferol vitamin D.

**Figure 7.**
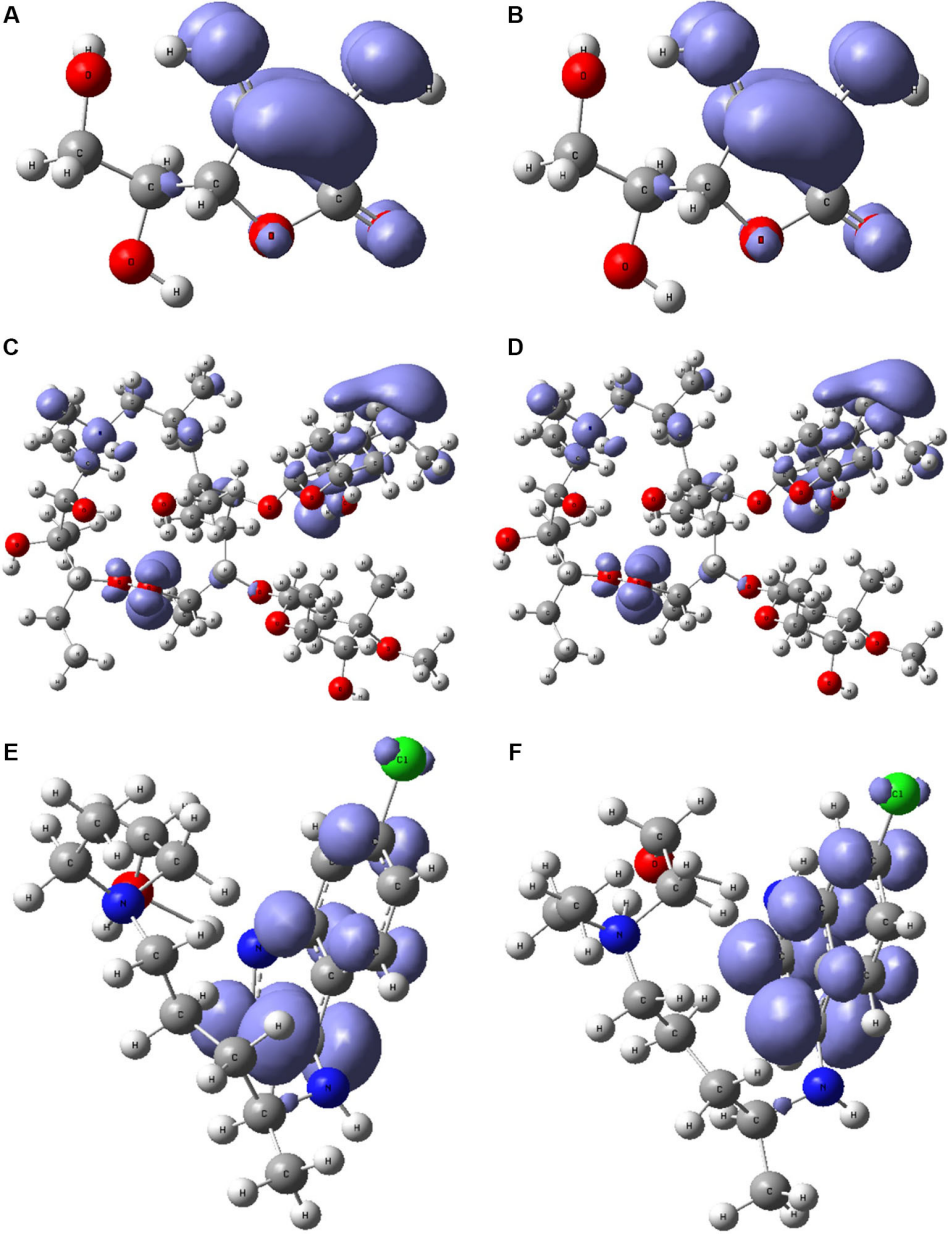
Local chemistry reactivity indices. A. Fukui function $f^{-}\left(r\right)\approx {\left|\text{HOMO}\right|}^{2}$ and B. $f^{+}\left(r\right)\approx {\left|\text{HOMO}\right|}^{2}$ related with ascorbic acid vitamin C. C. Fukui function $f^{-}\left(r\right)\approx {\left|\text{HOMO}\right|}^{2}$ and D. $f^{+}\left(r\right)\approx {\left|\text{HOMO}\right|}^{2}$ related with azithromycin. E. Fukui function $f^{-}\left(r\right)\approx {\left|\text{HOMO}\right|}^{2}$ and F. $f^{+}\left(r\right)\approx {\left|\text{HOMO}\right|}^{2}$ associated with hydroxychloroquine.

Figure 6 and Figure 7 show the Fukui functions $f^{-}\left(r\right)\approx {\left|\text{HOMO}\right|}^{2}$ and $f^{+}\left(r\right)\approx {\left|\text{HOMO}\right|}^{2}$ for the compounds selected. The Fukui function $f^{-}\left(r\right)\approx {\left|\text{HOMO}\right|}^{2}$ is associated with the susceptibility of a site to being attacked by electrophilic species, while $f^{+}\left(r\right)\approx {\left|\text{HOMO}\right|}^{2}$ represents the susceptibility of a site to being attacked by nucleophilic species. In these figures, we can see the functions of Fukui (-) and (+) on the identical zones in the molecular structure. These can be related to the stabilization process on the active site, showing the zones associated with global reactivity indices like electrophilicity and softness. These reactivity parameters can help understand the destabilization process and the bond formation (-H bonds) in non-covalent interactions. All these outcomes can be useful for the rapid assessment of the currently available antiviral drugs used for treating COVID-19 patients, which is crucial at this time of crisis, as well as for discovering newer drugs.

## Conclusions

In this study, a number of ligands related to the treatment of COVID-19 and used as inhibitors of SARS-CoV-2 virus, such as abacavir, acyclovir, amprenavir, ascorbic acid vitamin C, azithromycin, baloxavir, boceprevir, cholecalciferol vitamin D, cidofovir, edoxudine, emtricitabine, hydroxychloroquine and remdesivir, have been analyzed by molecular docking, molecular quantum similarity and chemical reactivity indices to study their active site stabilization interactions from a structural and electronic point of view.

In this study, to develop the docking experiments, some compounds were selected as references, these compounds are frequently related with the treatment of COVID-19. Some important anti-polymerase drugs were tested that are currently in clinical trials or on the market to stop viral infection on an emergency basis. The docking interaction for remdesivir, cholecalciferol vitamin D, azithromycin and ascorbic acid have shown good interaction (-H bonds) in the active site. The main idea is to extrapolate these outcomes and find novel insights into inhibitors for COVID-19.

The analysis of molecular quantum similarity indices on inhibitors showed high differences from a structural point of view. However, they are quite similar in their electronic density, obtaining the highest values in the electronic similarity index. Global and local chemical reactivity indices were calculated. These indices allow for the identification of the zones of chemical reactivity involved in the non-covalent stabilization of these inhibitors on the active site. Moreover, new outcomes about compounds such as abacavir, which is used to treat HIV infection, were shown and some studies have shown their relationship with COVID-19. In addition, it sheds light on the use of novel ligands for the treatment of this challenging disease that has claimed millions of lives worldwide.

## Data availability

### Underlying data

Protein Data Bank: SARS-Cov-2 RNA-dependent RNA polymerase in complex with cofactors. Accession number 6M71; https://identifiers.org/pdb:6M71

Harvard Dataverse: New insights in series of ligands used as SARS-CoV-2 virus inhibitors within molecular docking, molecular quantum similarity, and chemical reactivity indices frameworks. https://doi.org/10.7910/DVN/MSIGUS. ^51^

Data are available under the terms of the Creative Commons Zero D. “No rights reserved” data waiver (CC0 1.0 Public domain dedication).
