# Supplementary material for: New findings on ligand series used as SARS-CoV-2 virus inhibitors within the frameworks of molecular docking, molecular quantum similarity and chemical reactivity indices
Source: F1000Res. 2023 Dec 11;11:914. Originally published 2022 Aug 9. [Version 3] doi: 10.12688/f1000research.123550.3 (PMC12754369; doi:10.12688/f1000research.123550.3)
Supplement: Peer review history

# Reviewer report: Reviewer response for version 3

**DOI:** 10.5256/f1000research.159813.r437259

**Published:** 2026-01-06

**Authors:** Naveen Shivalingegowda

**Affiliations:** 1 Department of Physics, JAIN (Deemed-to-be University), Bangalore, India

I have carefully gone through the manuscript of the article submitted for indexing. While the manuscript presents an extensive computational analysis combining molecular docking, molecular quantum similarity (MQS), and conceptual DFT-based reactivity descriptors, the study fails to deliver substantive new mechanistic or predictive insight into SARS-CoV-2 RdRp inhibition. The work is method-heavy but hypothesis-light, and suffers from over-interpretation of qualitative docking results, lack of validation, weak biological grounding, and questionable novelty. In its current form, the manuscript reads more as a retrospective theoretical exercise than a contribution capable of informing antiviral discovery or drug repurposing strategies. While the manuscript demonstrates computational competence, it falls short of scientific rigor, novelty, and translational relevance. Without substantial restructuring, validation, and hypothesis-driven analysis, the work remains a methodological compilation rather than a meaningful contribution to SARS-CoV-2 drug discovery.

 Further, to improve the quality of the manuscript, I would suggest the authors to:

 Introduce a clear hypothesis and decision framework

 Provide validated docking metrics and comparative rankings

 Integrate MD + MM-GBSA or FEP analysis

 Correlate MQS and DFT descriptors with experimental antiviral data

 Remove or critically contextualize clinically disproven compounds

 Demonstrate predictive or screening utility, not just post hoc description

Is the work clearly and accurately presented and does it cite the current literature?

Partly

If applicable, is the statistical analysis and its interpretation appropriate?

Not applicable

Are all the source data underlying the results available to ensure full reproducibility?

Partly

Is the study design appropriate and is the work technically sound?

Partly

Are the conclusions drawn adequately supported by the results?

No

Are sufficient details of methods and analysis provided to allow replication by others?

Partly

Reviewer Expertise:

Molecular Biology

I confirm that I have read this submission and believe that I have an appropriate level of expertise to confirm that it is of an acceptable scientific standard, however I have significant reservations, as outlined above.

# Reviewer report: Reviewer response for version 3

**DOI:** 10.5256/f1000research.159813.r437260

**Published:** 2025-12-30

**Authors:** Emmanuel Kitete Mulongo

**Affiliations:** 1 University of Kinshasa, Kinshasa, Congo

This study remains relevant because COVID-19 persists, despite the emergence of new variants, even though the disease has lost some of its virulence.

1. The authors would strengthen the introduction by adding some sensitive numbers of victims during COVID-19 in the world
2. The citing model chosen (Mayo Clinic) is missing some information
3. The receptor used in this study has 3 major chains.  Because no details have been shared in the process, the authors should indicate the chain to which the substrates that interacted belong.
4. The receptor should be validated using a plotting process (RAMACHADRAN or other)
5. Authors should provide the grid box coordinates
6. In the conclusion section, the authors are suggested to give future direction, especially regarding the repurposing of Abacavir

Is the work clearly and accurately presented and does it cite the current literature?

Yes

If applicable, is the statistical analysis and its interpretation appropriate?

I cannot comment. A qualified statistician is required.

Are all the source data underlying the results available to ensure full reproducibility?

Partly

Is the study design appropriate and is the work technically sound?

Yes

Are the conclusions drawn adequately supported by the results?

Yes

Are sufficient details of methods and analysis provided to allow replication by others?

Partly

Reviewer Expertise:

Phytochemistry, medicinal chemistry, and in silico research

I confirm that I have read this submission and believe that I have an appropriate level of expertise to confirm that it is of an acceptable scientific standard, however I have significant reservations, as outlined above.

# Reviewer report: Reviewer response for version 3

**DOI:** 10.5256/f1000research.159813.r437257

**Published:** 2025-12-30

**Authors:** M. Aravinthraj Aravinthraj

**Affiliations:** 1 Department of Physics, Sacred Heart College (Autonomous), Tirupattur, Tamil Nadu, India

1.The author can change the softness unit in Table 5 and its discussion from eV to eV⁻¹.

 2.For visual consistency, change the background in Figure 6(A) white.

 3.Explain the correlation between the obtained GCRD values and its influences on SARS-CoV-2 and COVID-19?

 4.Why are hydrogen bond values not shown in the presented docking diagrams?

 5.In Figures 1-5, the author can mention what does the blue color in panels B and D represent?

 6.The docking discussion focuses only on distances. So include binding affinity (docking score) or interaction energy values for each ligand to strengthen the interpretation.

 7.For clarity, author can provide 2D interaction diagrams to complement the 3D visualizations? Also highlight the key residues.

Is the work clearly and accurately presented and does it cite the current literature?

Partly

If applicable, is the statistical analysis and its interpretation appropriate?

I cannot comment. A qualified statistician is required.

Are all the source data underlying the results available to ensure full reproducibility?

Yes

Is the study design appropriate and is the work technically sound?

Yes

Are the conclusions drawn adequately supported by the results?

Partly

Are sufficient details of methods and analysis provided to allow replication by others?

Yes

Reviewer Expertise:

DFT, MD

I confirm that I have read this submission and believe that I have an appropriate level of expertise to confirm that it is of an acceptable scientific standard, however I have significant reservations, as outlined above.

# Reviewer report: Reviewer response for version 3

**DOI:** 10.5256/f1000research.159813.r229291

**Published:** 2024-02-16

**Authors:** Yogesh Kumar

**Affiliations:** 1 University Medical Centre Hamburg-Eppendorf, Hamburg, Germany

Comments:

  

1. Why did the author draw the molecule structure when these structures are available in the PubChem database?
2. How many molecules author download or design?
3. Why did the author only select several molecules for this study?
4. What is the biological role of each selected molecule here?
5. There is no need to explain the Quantum similarity measure so deeply here, this method is well established and you have only used this method for your molecules.
6. The conclusion is not clear in the manuscript and I don’t understand what the message of the author here is.

Is the work clearly and accurately presented and does it cite the current literature?

No

If applicable, is the statistical analysis and its interpretation appropriate?

Yes

Are all the source data underlying the results available to ensure full reproducibility?

Yes

Is the study design appropriate and is the work technically sound?

No

Are the conclusions drawn adequately supported by the results?

No

Are sufficient details of methods and analysis provided to allow replication by others?

Yes

Reviewer Expertise:

Bioinformatics, Immunology, Data Sciences, Computational Biology

I confirm that I have read this submission and believe that I have an appropriate level of expertise to confirm that it is of an acceptable scientific standard, however I have significant reservations, as outlined above.

# Response

**Published:** 2024-02-16

**Authors:** Alejandro Morales-Bayuelo

**Affiliations:** Universidad del Sinú, Colombia

1. Why did the author draw the molecule structure when these structures are available in the PubChem database?

Answer: yes

1. How many molecules author download or design?

Answer: 13

1. Why did the author only select several molecules for this study?

Answer: Taking account, the biological activity and potential use for the CoVID-19 treatment.

1. What is the biological role of each selected molecule here?

Answer: The main idea is to show the interaction related with these ligands with CoVID-19 protein related and in these sense can be used as potential drug related with the CoVID-19 treatment.

1. There is no need to explain the Quantum similarity measure so deeply here, this method is well established, and you have only used this method for your molecules.

Answer: ok

1. The conclusion is not clear in the manuscript, and I don’t understand what the message of the author here is.

Answer: The development of new ligands or drugs for COVID-19 is crucial for several reasons:

1. **Variability of the Virus:** Viruses, including SARS-CoV-2 (the virus that causes COVID-19), can undergo mutations, leading to the emergence of new variants. Some variants may affect the virus's susceptibility to existing treatments. Developing new ligands or drugs allows researchers to stay ahead of these changes and ensure the effectiveness of therapeutic interventions.
2. **Enhanced Treatment Options:** Having a diverse range of drugs or ligands allows for more treatment options. Different drugs may target the virus through various mechanisms, increasing the chances of finding effective treatments for different patient populations or stages of the disease.
3. **Combating Drug Resistance:** As with any infectious agent, there is a risk of the virus developing resistance to existing treatments over time. By continually developing new ligands or drugs, researchers can stay ahead of potential drug resistance and provide alternative options for managing the infection.
4. **Optimizing Therapeutic Efficacy:** Developing new ligands allows scientists to optimize the efficacy and safety profile of treatments. This can lead to drugs that are more potent, have fewer side effects, and are better suited for certain patient populations.
5. **Addressing Unmet Needs:** The global impact of the COVID-19 pandemic has highlighted the need for effective and accessible treatments. Developing new ligands can help address unmet medical needs, especially in cases where existing treatments may not be sufficient or widely available.
6. **Preparation for Future Pandemics:** Investing in the development of antiviral drugs and ligands not only helps in the current fight against COVID-19 but also prepares us for potential future pandemics caused by novel viruses. The knowledge gained from developing treatments for one virus can inform strategies for addressing others.

In summary, developing new ligands for COVID-19 is crucial for staying ahead of viral mutations, expanding treatment options, combating drug resistance, optimizing therapeutic efficacy, addressing unmet needs, and preparing for future pandemics.

# Reviewer report: Reviewer response for version 3

**DOI:** 10.5256/f1000research.159813.r229107

**Published:** 2023-12-18

**Authors:** Truong Tan Trung

**Affiliations:** 1 Dong Nai Technology University, Vietnam, Vietnam

I agree with the author's revised manuscript.

Is the work clearly and accurately presented and does it cite the current literature?

Partly

If applicable, is the statistical analysis and its interpretation appropriate?

I cannot comment. A qualified statistician is required.

Are all the source data underlying the results available to ensure full reproducibility?

Yes

Is the study design appropriate and is the work technically sound?

Yes

Are the conclusions drawn adequately supported by the results?

Partly

Are sufficient details of methods and analysis provided to allow replication by others?

Yes

Reviewer Expertise:

Computational chemistry; DFT; Nanotechnology; Docking simulation

I confirm that I have read this submission and believe that I have an appropriate level of expertise to confirm that it is of an acceptable scientific standard.

# Reviewer report: Reviewer response for version 2

**DOI:** 10.5256/f1000research.153172.r195605

**Published:** 2023-08-22

**Authors:** Truong Tan Trung

**Affiliations:** 1 Dong Nai Technology University, Vietnam, Vietnam

1. Page 7: fk+ not fk- is for nucleophilic attack.
2. Page 17: Euclidean distance of 16.8664 (Table 4);  not 22.2556 (Emitri/Acyc).
3. Page 18: softness S= 0.3916, not 1.2136; electrophilicity =1.2136, not 1.6253; seen in Table 5.

Is the work clearly and accurately presented and does it cite the current literature?

Partly

If applicable, is the statistical analysis and its interpretation appropriate?

I cannot comment. A qualified statistician is required.

Are all the source data underlying the results available to ensure full reproducibility?

Yes

Is the study design appropriate and is the work technically sound?

Yes

Are the conclusions drawn adequately supported by the results?

Partly

Are sufficient details of methods and analysis provided to allow replication by others?

Yes

Reviewer Expertise:

Computational chemistry; DFT; Nanotechnology; Docking simulation

I confirm that I have read this submission and believe that I have an appropriate level of expertise to confirm that it is of an acceptable scientific standard.

# Response

**Published:** 2023-12-05

**Authors:** Alejandro Morales-Bayuelo

**Affiliations:** Universidad del Sinú, Colombia

1. Page 7: fk+ not fk- is for nucleophilic attack. Answer: Done
2. Page 17: Euclidean distance of 16.8664 (Table 4);  not 22.2556 (Emitri/Acyc). Answer: Done.
3. Page 18: softness S= 0.3916, not 1.2136; electrophilicity =1.2136, not 1.6253; seen in Table 5. Answer: Done

# Reviewer report: Reviewer response for version 1

**DOI:** 10.5256/f1000research.135665.r185712

**Published:** 2023-07-17

**Authors:** Truong Tan Trung

**Affiliations:** 1 Dong Nai Technology University, Vietnam, Vietnam

The work is of good importance, however, there are various things that need to be addressed and the amount of work is not sufficient. Without addressing them, I would not recommend the indexing of this manuscript.

1. What are the novelties of the manuscript? What’s new in this manuscript compared to similar papers?
2. The introduction section should be written in a higher quality way, i.e. more up-to-date references. The research gap should be delivered more clearly with the directed necessity for the novelties of the work.
3. Have the selected compounds been reported as potential ligands in the treatment of SARS-CoV-2?
4. The position of reference 3 was not defined in the manuscript.
5. “Ascorbic acid vitamin C” should be written as ascorbic acid (vitamin C). Same for cholecalciferol vitamin D “cholecalciferol (Vitamin D3)”.
6. Page 6: I think it should be "fr+ " is for nucleophilic attack.
7. The sentence “The lowest values...of 7.1944” at the bottom of page 10 is confusing, rephrase it (missing value Euclidean distance of 8.6174 of Boce/Azythr).
8. Page 11, the value of Euclidean distance of edox/acyc is 22.2556. It is different from the value shown in Table 4 (16.8664). The author should reconsider.
9. Pages 11 and 16, character symbol of unknown, e.g. chemical hardness.
10. Please add the HOMO and LUMO energy values in Table 5. Furthermore, the band energy gap of compounds in the binding pocket of the receptor. This will depict the reactivity with the receptor.
11. Page 16, the value of softness (S=1.2136 eV) and electrophilicity (w=1.6253) of Azithromycin is different from the values shown in Table 5. Carefully check the manuscript for similar errors.
12. Page 3, need ≥ 100 ns molecular dynamics.
13. About the docking results: The author clearly presents the results obtained from the docking in the form of data in the table, which is used as evidence for discussion.

Is the work clearly and accurately presented and does it cite the current literature?

Partly

If applicable, is the statistical analysis and its interpretation appropriate?

I cannot comment. A qualified statistician is required.

Are all the source data underlying the results available to ensure full reproducibility?

Yes

Is the study design appropriate and is the work technically sound?

Yes

Are the conclusions drawn adequately supported by the results?

Partly

Are sufficient details of methods and analysis provided to allow replication by others?

Yes

Reviewer Expertise:

Computational chemistry; DFT; Nanotechnology; Docking simulation

I confirm that I have read this submission and believe that I have an appropriate level of expertise to confirm that it is of an acceptable scientific standard, however I have significant reservations, as outlined above.

# Response

**Published:** 2023-07-17

**Authors:** Alejandro Morales-Bayuelo

**Affiliations:** Universidad del Sinú, Colombia

1. What are the novelties of the manuscript? What’s new in this manuscript compared to similar papers?

**Answer:** There is currently no effective treatment for Covid-19. Therefore, there are a series of drugs such as those listed in this article that have been used for the treatment of this disease, however they have not been associated as solutions for covid-19. That is why carrying out studies that show that the drugs used are directly related to the Covid-19 proteins is pertinent.

 2. The introduction section should be written in a higher quality way, i.e. more up-to-date references. The research gap should be delivered more clearly with the directed necessity for the novelties of the work.

**Answer:** Done.

 3. Have the selected compounds been reported as potential ligands in the treatment of SARS-CoV-2?

**Answer:** The molecular datasets used for the docking study were reported by Sipos et al.4 , Székely et al.5 , Lougheed [6] , Chapman [7] and finally Pato [8] (see Table 1–Table 6). To select this molecular dataset, the structural diversity and uniform distribution of IC50 was taken into account. Logarithmic IC _50_ (μM) (PIC _50_=−log IC _50_) was employed as the dependent variable instead of IC _50_. All molecular set, has been used as treatment for the Covid-19.

 4. The position of reference 3 was not defined in the manuscript.

**Answer:** Pkn A reported by Sipos [3].

 5. “Ascorbic acid vitamin C” should be written as ascorbic acid (vitamin C). Same for cholecalciferol vitamin D “cholecalciferol (Vitamin D3)”.

**Answer:** Done.

 6. Page 6: I think it should be "fr+ " is for nucleophilic attack.

**Answer:** Done.

 7. The sentence “The lowest values...of 7.1944” at the bottom of page 10 is confusing, rephrase it (missing value Euclidean distance of 8.6174 of Boce/Azythr).

**Answer:** Done.

 8. Page 11, the value of Euclidean distance of edox/acyc is 22.2556. It is different from the value shown in Table 4 (16.8664). The author should reconsider.

**Answer:** Done.

 9. Pages 11 and 16, character symbol of unknown, e.g. chemical hardness.

**Answer:** The Chemical Hardness (Ƞ) has been symbolized using the standard Density Functional Framework.

 10. Please add the HOMO and LUMO energy values in Table 5. Furthermore, the band energy gap of compounds in the binding pocket of the receptor. This will depict the reactivity with the receptor.

**Answer:** In the Supporting Information has been added the output files used for the calculations of the reactivity indexes for each compound.

 11. Page 16, the value of softness (S=1.2136 eV) and electrophilicity (w=1.6253) of Azithromycin is different from the values shown in Table 5. Carefully check the manuscript for similar errors.

**Answer:** Done.

 12. Page 3, need ≥ 100 ns molecular dynamics.

**Answer:** In this study, the best predictions of the poses were predicted by 30ns molecular dynamic calculations. This values by 30ns for molecular dynamic has been used by our research group in several publication taking account the docking values.

 13. About the docking results: The author clearly presents the results obtained from the docking in the form of data in the table, which is used as evidence for discussion.

**Answer:** ok.
